# Rhythmic network activity in human brain slices: variability, mechanisms, and translational insights

**DOI:** 10.3389/fnsyn.2026.1798456

**Published:** 2026-03-09

**Authors:** Danqing Yang, Dirk Feldmeyer

**Affiliations:** 1 Institute of Neuroscience and Medicine 10, Research Center Juelich, Juelich, Germany; 2 Department of Psychiatry, Psychotherapy and Psychosomatics, RWTH Aachen University Hospital, Aachen, Germany; 3 Jülich-Aachen Research Alliance, Translational Brain Medicine (JARA Brain), Aachen, Germany

**Keywords:** human brain slice, network oscillations, rhythmic activity, interneurons, ionic mechanisms

## Abstract

*In vitro* maintained human brain slices provide a unique experimental platform for investigating rhythmic neuronal network activity, bridging the gap between animal models and clinical studies. A wide range of spontaneous and induced oscillatory activities has been described in human brain slices. However, their occurrence and characteristics are strongly shaped by methodological determinants spanning tissue origin, slice preparation, recording conditions, and induction strategies. This has been shown to have a profound impact on the reproducibility and interpretation of oscillatory dynamics. This review synthesizes current evidence on rhythmic network activity in acute human brain slices, with a particular emphasis on how methodological determinants interact with intrinsic circuit properties to generate oscillatory dynamics. We discuss how different experimental manipulations influence oscillation frequency, stability, and spatial organization. We further examine the cellular and circuit mechanisms underlying rhythmic activity, highlighting the roles of excitatory–inhibitory balance, synaptic dynamics, neuromodulatory influences, and distinct interneuron populations. Finally, we consider how oscillatory patterns differ across disease contexts, particularly epilepsy and tumor-associated cortex, and discuss the translational value and limitations of human brain slices for linking microcircuit mechanisms to pathological and functional brain states.

## Introduction

Rhythmic neuronal network activity is a basic feature of neuronal networks in the human brain. Neural oscillations occur across a wide range of timescales, from infra-slow oscillations to fast rhythms such as γ- and higher-frequency activity (Buzsaki and Draguhn, 2004; Steriade, 2006). Together, these oscillations support communication in neuronal networks, influence synaptic integration and organize information processing within and across brain regions (Fries, 2005; Siegel et al., 2012). Neuronal oscillations are tightly linked to brain states such as cognition, attention, memory formation, and sleep–wake regulation. Aberrant oscillatory activity is increasingly recognized not only as a marker but also as a potential driver of brain pathology. For instance, epilepsy is characterized by pathological hypersynchrony and abnormal rhythmic discharges, whereas neurodegenerative disorders such as Alzheimer’s disease are associated with marked alterations in γ- and θ-rhythms (Goodman et al., 2019; Jiruska et al., 2013; Palop and Mucke, 2016). More broadly, disruptions of rhythmic brain activity are commonly observed in neurological and psychiatric disorders, such as epilepsy, schizophrenia, and neurodegenerative diseases (Ploner et al., 2006; Uhlhaas and Singer, 2010; Voytek and Knight, 2015; Wang, 2010). Importantly, alterations in oscillatory patterns often emerge before overt structural degeneration or the appearance of clinical symptoms, suggesting that rhythmic activity may serve as an early and sensitive indicator of circuit dysfunction. These observations highlight oscillatory dynamics as both functional substrates of healthy brain operation and sensitive indicators of pathological circuit states.

At the cellular and circuit level, oscillations emerge from coordinated interactions between excitatory pyramidal neurons and inhibitory interneuron populations. Their expression is shaped by intrinsic membrane properties, synaptic kinetics, network connectivity, and neuromodulatory influences. Slow and infra-slow oscillations are typically associated with global network states and up–down transitions, whereas faster rhythms such as θ and γ rhythms allow temporally precise synchronization and information processing (Klausberger et al., 2004; Steriade et al., 2001; Womelsdorf et al., 2007). Cross-frequency coupling further links activity across timescales, providing a flexible mechanism for coordinating neuronal ensembles (Canolty and Knight, 2010).

Despite their importance, much of our current understanding of neuronal oscillations comes from studies in animal models, particularly rodents, using *in vivo* recordings and *in vitro* slice preparations. While these approaches have provided valuable mechanistic insights, accumulating evidence indicates notable differences between rodent and human brains in cortical organization, synaptic physiology, interneuron composition, and network dynamics (Beaulieu-Laroche et al., 2018; Campagnola et al., 2022; Gidon et al., 2020; Hodge et al., 2019; Hunt et al., 2023; Wilbers et al., 2023; Yang et al., 2024). Such differences complicate the direct translation of findings from animals to humans and underscore the need for experimental approaches that allow direct investigation of rhythmic activity in human neuronal circuits.

Ηuman brain slices maintained *in vitro* provide a unique opportunity to bridge the gap between animal electrophysiology and human physiology and pathophysiology. Brain slices prepared from neurosurgical resections, typically performed for epilepsy or tumor treatment, preserve local microcircuit architecture to a large degree and enable direct recordings from human neurons and networks. In contrast to animal models, human slices retain species-specific synaptic properties, interneuron composition, and laminar organization, allowing direct investigation of human-relevant mechanisms of rhythm generation (Beaulieu-Laroche et al., 2018; Berg et al., 2021; Peng et al., 2024). Importantly, human brain slices offer advantages over animal models. These advantages are twofold: firstly, they provide an experimental setting in which network dynamics and pharmacological responses can be assessed directly in human neuronal circuits; secondly they allow direct examination of disease-relevant tissue, including epileptic foci in the cortex, hippocampal sclerosis, and brain tissue adjacent to tumors. Together, these features establish human brain slices as a valuable platform for translational studies, such as the evaluation of antiepileptic or neuromodulatory compounds in human-specific networks.

Using techniques such as extracellular field potential recordings, multi-electrode arrays, and intracellular patch-clamp techniques, a wide spectrum of oscillatory phenomena has been documented in human cortical and hippocampal slices. Both spontaneous and experimentally induced oscillations have been reported, with their frequency, spatial extent, and pharmacological sensitivity varying substantially across studies, brain regions, and pathological contexts (Alvarado-Rojas et al., 2015; Avoli et al., 1994; Roopun et al., 2010; Toth et al., 2018; Yang et al., 2024). Such variability likely reflects a combination of intrinsic biological diversity and methodological heterogeneity. This highlights important limitations of human resected tissue as an experimental platform: Access to human tissue is inherently restricted and ethically constrained, and truly “healthy” control tissue is rarely available. In addition, variability in tissue origin, patient history, medication, and disease state introduces substantial confounds. Experimental procedures including tissue transport, slicing methods, recovery protocols, and recording conditions such as the composition of intra- and extracellular solutions further influence neuronal viability and network excitability. Consequently, oscillatory activity observed in human brain slices is highly sensitive to methodological parameters. This complicates cross-study comparisons and raises important questions regarding reproducibility and physiological interpretation. Separating biologically meaningful rhythms from preparation-induced phenomena is essential for translating findings from human slices to clinical and cognitive neuroscience.

In this review, we provide a comprehensive synthesis of current knowledge on rhythmic network activity in human brain slices, with particular emphasis on sources of variability, neural modulation and circuit mechanisms. We discuss how methodological procedures such as slice preparation and maintenance, recording strategies, and approaches used to elicit oscillations influence the expression of network rhythms. We then examine the cellular and circuit processes that support oscillatory activity, emphasizing the contributions of specific interneuron populations, excitatory–inhibitory interactions, and synaptic dynamics. Based on this mechanistic context, we compare oscillatory patterns across disease states and species to highlight features that appear specific to the human brain and to identify gaps in current translational approaches. Overall, this review aims to describe a practical approach for interpreting oscillatory activity in human brain slices and to support more consistent and translationally informative experimental strategies.

## Methodological variability and its impact on rhythmic activity

### Human brain slice preparation and recording methodologies

The majority of rhythmic activity in human neurons described in the literature has been recorded in acute human brain slices, that were typically prepared within a narrow time window following neurosurgical resection (Table 1). Acute slices preserve much of the normal microcircuit architecture, laminar organization, and synaptic connectivity necessary for the emergence of network-level rhythms. Complementarily, organotypic human slice cultures provide a powerful platform for long-term experimental access, enabling viral-mediated genetic manipulation, stable fluorescent labeling, and repeated imaging over extended time scales (Bak et al., 2024; Ravi et al., 2019; Schwarz et al., 2019). However, extended *in vitro* maintenance can be accompanied by gradual circuit reconfiguration and homeostatic adjustments in synaptic transmission that may alter network dynamics compared to acute slice preparations. Therefore, the present review focuses on rhythmic activity characterized in *acute* human brain slice recordings, which constitute the prevalent experimental system used in the recent literature.

**Table 1:** *Ex-vivo* network oscillations recorded in human acute brain slices.

Oscillation type (frequency)	Brain region	Pathology	Induction	Slice thickness (μm)	aCSF ionic composition			Recording temperature and chamber	Modulation	References
					Mg^2+^ (mM)	Ca^2+^ (mM)	K^+^ (mM)			
Infra-slow oscillations										
Synchronous field potential (~0.1 Hz)	Neocortex L4-5	Epilepsy; epileptic cortex	4-AP application	500–600	2	1.8	2	34–36 °C, interface chamber	Blocked by bicuculline; modulated by CNQX&CPP	Avoli et al. (1994)
Interictal-like discharges (IID, 0.05–0.2 Hz)	Neocortex L4-6	Epilepsy; epileptic cortex	Spontaneous	350	1	1	3.5	35–37 °C, interface chamber	—	Toth et al. (2018)
Up states (0.05–0.5 Hz)	Neocortex L2-3 INs/PCs	Epilepsy/tumor; distant from foci	Spontaneous	300	1	2	2.5	31–33 °C, submerged chamber	—	Yang et al. (2024)
Slow oscillations										
Large rhythmic depolarizations (LRDs, 0.1–0.7 Hz)	Neocortex L2-3 INs/PCs	Epilepsy/tumor; distant from foci	Spontaneous/ NE application	300	1	2	2.5	31–33 °C, submerged chamber	Blocked by TTX, CNQX and ACh; enhanced by NE	Yang et al. (2024)
Slow wave activity (0.2–1 Hz)	Temporal cortex	Epilepsy; distant from foci	Spontaneous	400	1	1	4	34.5–36 °C, interface chamber	Modulated by bicuculline	Covelo et al. (2025)
Interictal epileptiform discharges (0.1–1.5 Hz)	Hippocampus CA2 and subiculum	Epilepsy; epileptic hippocampus	Spontaneous	400	2	2	4	35–37 °C, interface chamber	—	Wittner et al. (2009)
Interictal-like slow rhythmic field potentials (~0.4 Hz)	Human lateral amygdala	Epilepsy; non-sclerotic amygdala	Spontaneous	400–500	1.3	2	4	33 °C, submerged chamber	Blocked by CNQX and bicuculline	Graebenitz et al. (2011)
Synchronous population activity (SPA, 0.3–1.6 Hz)	Neocortex L2-3	Epilepsy /tumor resected tissue with/without seizure	Spontaneous	350–500	1	1	3.5	35–37 °C, interface chamber	Blocked by NBQX and bicuculline; suppressed by APV	Toth et al. (2018) and Bod et al. (2023)
Spontaneous sharp waves (0.07–1.8 Hz)	Neocortex	Epilepsy; epileptic cortex	Spontaneous	400–500	1	1	4	28 °C, submerged chamber	Blocked by CNQX and bicuculline	Kohling et al. (1998)
δ oscillations										
Interictal discharges (IID, 0.6–1.8 Hz)	Subiculum	Epilepsy; with hippocampal sclerosis	Spontaneous	400	2	2	4	37 °C, interface chamber	—	Alvarado-Rojas et al. (2015)
Interictal-like activity (1.2 ± 0.6 Hz)	Subiculum	Epilepsy; with hippocampal sclerosis	Spontaneous	400	2	2	4	35–37 °C, interface chamber	Blocked by NBQX+APV and bicuculline	Cohen et al. (2002)
Preictal discharges (PID, 0.5–3 Hz)	Subiculum	Epilepsy; with hippocampal sclerosis	proconvulsant ACSF	400	0.5	0.5	6–8	37 °C, interface chamber	—	Alvarado-Rojas et al. (2015)
Low-frequency rhythms (1–3 Hz)	Temporal cortex and hippocampus	Epilepsy; epileptic cortex	Spontaneous	750	2	2	5	35 °C, interface chamber	Blocked by TTX and bicuculline	Schwartzkroin and Knowles (1984) and Schwartzkroin and Haglund (1986)
θ–γ oscillations										
θ–γ coupled oscillations (θ: 4–7 Hz; γ: 30–100 Hz)	Temporal cortex L2/3 INs and PCs	Epilepsy; seizure onset zone	Spontaneous	450	1	1.2	3	34–36 °C, interface chamber	Persists under synaptic blockade (AMPA, NMDA, GABA blockers), blocked by gap-junction blocker (carbenoxolone)	Roopun et al. (2010)
Very fast oscillations (VFOs, >80 Hz)	Temporal neocortex L2/3 and L5/6	Epilepsy; distant from foci	Kainate + carbachol application; electrical stimulation	500	1	1	4	36 °C, submerged chamber	Blocked by atropine; suppressed by phenytoin	Florez et al. (2015)

**Figure 1:**
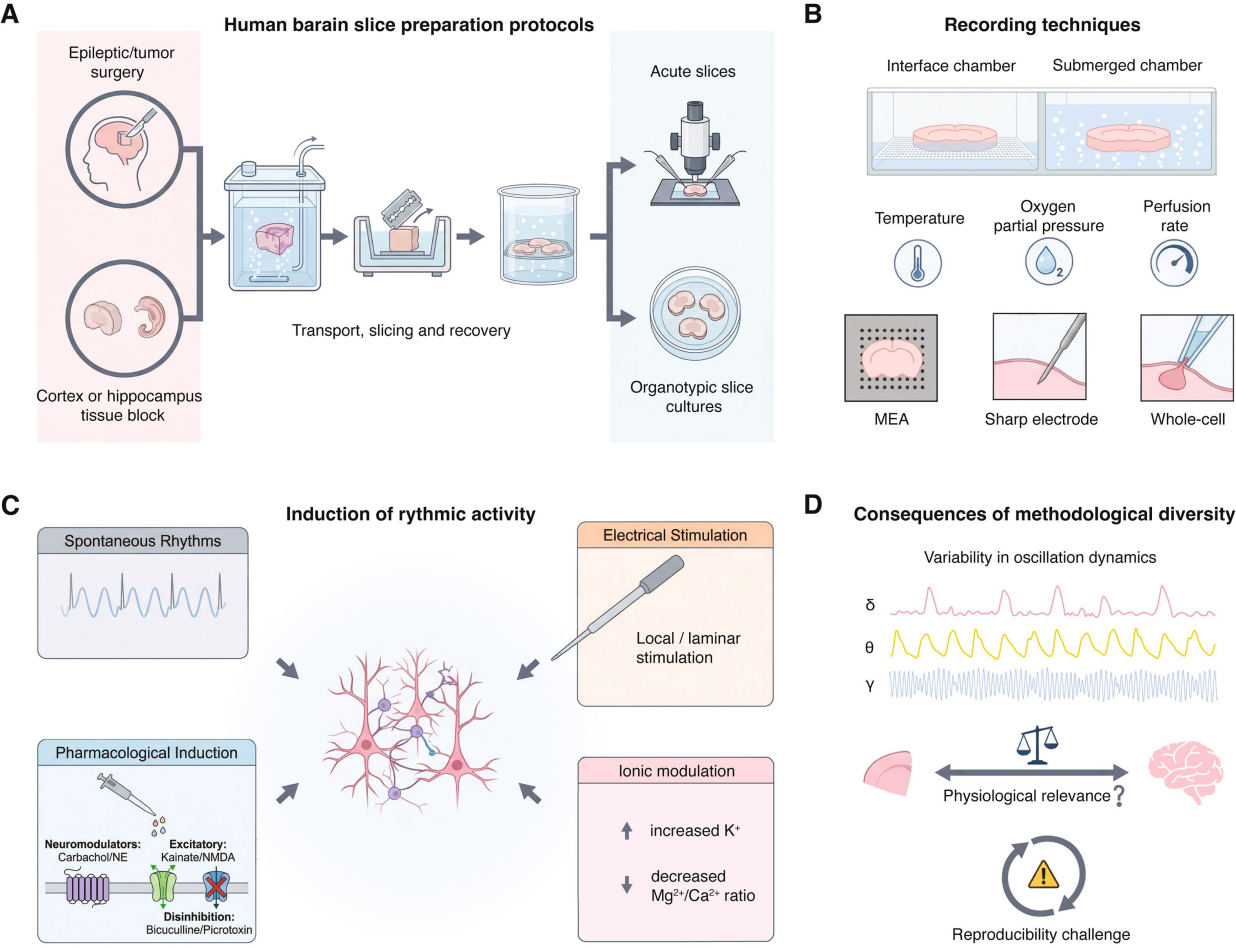
Methodological determinants and translational implications of rhythmic network activity in human brain slices. **(A)** Human cortical and hippocampal tissue is typically obtained from neurosurgical resections (e.g., epilepsy or tumor surgery) or, less frequently, from post-mortem donors. Variability in tissue origin, transport conditions, slicing procedures, and recovery protocols critically shapes neuronal viability and network excitability. **(B)** Rhythmic activity can be studied using submerged or interface recording chambers combined with extracellular field recordings, multi-electrode arrays, or intracellular patch-clamp techniques. Parameters such as oxygenation, temperature, and perfusion rate strongly influence the emergence and stability of oscillations. **(C)** Network oscillations can arise spontaneously or be induced by electrical stimulation, pharmacological manipulation (e.g., cholinergic or glutamatergic activation, GABAergic blockade), or ionic modulation of the extracellular milieu. These rhythms are shaped by interactions between excitatory pyramidal neurons and diverse interneuron subtypes, as well as by synaptic and circuit-level dynamics. **(D)** Differences in experimental approaches lead to substantial variability in oscillation frequency, amplitude, and temporal structure, complicating cross-study comparisons and limiting reproducibility. Consequently, rhythmic activity in human brain slices requires cautious interpretation and standardized experimental frameworks before cellular and circuit mechanisms can be meaningfully related to network-level dynamics and clinically relevant phenotypes.

As schematically illustrated in Figure 1, variability is introduced at multiple experimental stages, including tissue origin, slice preparation and maintenance, recording configurations, and the strategies used to elicit or modulate oscillatory activity. Human brain tissue is mostly obtained from epilepsy surgery or tumor resections, with “control” tissue typically originating from areas distant from seizure foci or tumor margins (Figure 1A). As summarized in Table 1, infra-slow and slow oscillations, including interictal-like discharges, are predominantly reported in epileptic neocortex and are most frequently recorded from deep layers (L4–6) neurons. In contrast, slow rhythmic activities observed in tissue distant from the epileptogenic focus, such as spontaneous up states and large rhythmic depolarizations (LRDs), are mainly described in superficial layers (L2/3). This laminar dissociation is consistent with clinical evidence indicating that seizure initiation most often involves deep cortical layers (Bourdillon et al., 2024; Jarre et al., 2017). Although tumor-resected human brain tissue is frequently used to demonstrate that observed rhythmic activities are not directly related to *in vivo* epileptic seizures (Toth et al., 2018; Yang et al., 2024), it is important to note that most brain tumors are themselves associated with seizures, and ictal discharges can propagate beyond the seizure onset zone to involve surrounding brain tissue (Bourdillon et al., 2024; van Breemen et al., 2007).

Transport and slicing procedures critically influence neuronal viability and network excitability. Rapid transfer of tissue in oxygenated, ice-cold solutions such as sucrose- or N-methyl-D-glucamine (NMDG)-based cutting solution designed to minimize excitotoxicity and metabolic stress, is essential for preserving rhythmic network activity. In addition, optimized recovery protocols, including controlled rewarming, prolonged incubation in oxygenated artificial cerebrospinal fluid, and gradual restoration of physiological ionic conditions, are critical for the re-emergence of spontaneous oscillations (Figure 1A) (Hajos et al., 2009; Perumal and Sah, 2022; Ting et al., 2018). Differences in tissue handling, such as transport duration, can strongly influence whether spontaneous oscillations are retained. Experimental observations indicate that spontaneous low-frequency rhythm are markedly reduced after prolonged transport or suboptimal recovery, even when neuronal survival appears intact at the single-cell level (Qi et al., 2026). This highlights that the absence of rhythmic activity may reflect methodological suppression rather than an intrinsic lack of oscillatory capability. Thicker slices are generally preferred for preserving network oscillations, studies of *ex vivo* human brain slice most commonly use a slice thickness of around 400 μm, with an overall range of 300–750 μm (Table 1). In contrast, thinner slices are more commonly used for detailed cellular-level recordings aimed at characterizing single-cell properties (Berg et al., 2021; Hodge et al., 2019; Molnar et al., 2008).

Another influential methodological variable shaping rhythmic activity is the choice of recording chamber (Figure 1B). As evident from Table 1, interface chambers are frequently associated with the detection of robust slow oscillations, interictal discharges, and large-scale population rhythms across neocortical and hippocampal regions. Interface chambers provide superior oxygenation by exposing the slice surface to humidified carbogen, which is particularly important for thick human slices (400–750 μm). In contrast, submerged chambers, although advantageous for visualized patch-clamp recordings, often require thinner slices and higher perfusion rates, conditions that may suppress large-scale synchronization (Maier et al., 2009). Moreover, different recording techniques preferentially measure distinct aspects of rhythmic activity. Extracellular field recordings and multi-electrode arrays are well suited for detecting spatially extended, synchronous oscillations, such as infra-slow rhythms, δ activity, or θ-γ coupling. In contrast, whole-cell recordings reveal subthreshold oscillations, up states, and LRDs at the level of individual neurons, sometimes in the absence of a clear field potential correlate. These different methodological approaches can result into divergent interpretations of whether a rhythm reflects a global network phenomenon or a localized, cell-type–specific process. Therefore, comparisons across studies employing different recording modalities must be made with caution. Recording temperature varies substantially across studies, ranging from ~28 °C to near-physiological values of 35–37 °C (Table 1). Changes in temperature modulate neuronal excitability primarily through effects on temperature-sensitive ion channels and membrane kinetics, thereby altering synaptic integration and intrinsic firing dynamics. In hippocampal neurons, reductions in temperature slow down voltage- and ligand-gated conductances and prolong membrane time constants, which can favor sustained depolarizations and slow rhythmic activity at both the single-cell and network levels (de la Pena et al., 2012; Thompson et al., 1985). Taken together, suboptimal control of parameters during recording can lead to unstable or transient oscillations, complicating a mechanistic interpretation.

### Induction of rhythmic activity

An important distinction is whether rhythms arise spontaneously or require induction. As summarized in Table 1, spontaneous infra-slow oscillations, and slow wave activity can occur following acute slice preparation under appropriate conditions. However, higher-frequency rhythms, including δ-, θ-, and γ-oscillations, are reported commonly for experimental conditions involving ionic modulation of the extracellular solution, or additional experimental induction, such as pharmacological manipulation or patterned stimulation (Figure 1C).

Electrical stimulation offers a controlled way of engaging network oscillations that may not emerge spontaneously. Local or laminar electrical stimulation has been shown to evoke γ-range activity through engagement of local inhibitory networks (Whittington et al., 1995), while repetitive or rhythmic stimulation protocols can engage endogenous oscillatory dynamics through phase locking and entrainment mechanisms (Herrmann et al., 2016; Thut et al., 2011). In human neocortical slices, such approaches have been used to probe network responsiveness and to facilitate the emergence of fast oscillatory activity under permissive conditions (Florez et al., 2015). Although less commonly employed than pharmacological approaches, electrical induction provides complementary insights into the latent oscillatory capacity of human circuits and their ability to support phase locking and synchronization.

Pharmacological manipulation is one of the most widely used experimental strategies for eliciting rhythmic activity in human brain slices. As summarized in Table 1, cholinergic agonists (e.g., carbachol) and noradrenergic modulation can induce slow oscillations, LRDs, or fast rhythmic activity by engaging neuromodulatory systems. Activation of glutamatergic receptors using kainate or N-methyl-D-aspartate (NMDA) receptor co-activation promotes γ-range or very fast oscillations, whereas a block of γ-aminobutyric acid type A (GABA_A_) receptor channels with bicuculline or picrotoxin typically induces hyperexcitability-driven rhythms resembling interictal discharges (Figure 1C). Importantly, oscillations induced by different pharmacological agents are not necessarily equivalent, even when they exhibit similar frequency bands.

Altering the extracellular ionic composition of the recording solution represents another powerful means of shaping network dynamics. Elevated extracellular K^+^ or reduced Mg^2+^ and Ca^2+^ concentrations are commonly associated with slow oscillations, preictal discharges, or low-frequency rhythmic activity. The substitution of the bath solution for one that more closely resembles the ionic composition of brain interstitial fluids resulted in the appearance of spontaneous rhythmic oscillations and the induction of cortical Up states similar to those observed *in vivo* (Sanchez-Vives and McCormick, 2000; Yang et al., 2024). This is because increasing the extracellular K^+^ concentration depolarizes the resting membrane potential and reduces the driving force for K^+^, thereby bringing large neuronal populations closer to firing threshold and enhancing network synchrony. In parallel, lowering the extracellular Mg^2+^ concentration relieves the voltage-dependent block of NMDA receptors, promoting sustained, regenerative depolarizations that support prolonged up states and slow oscillatory dynamics. A reduced extracellular Ca^2+^ concentration further increases neuronal excitability by decreasing surface charge screening and modifying synaptic release and short-term plasticity. Explicitly proconvulsant artificial cerebrospinal fluid (aCSF), combining very low Mg^2+^, low Ca^2+^, and supra-physiological high K^+^, has been used to induce preictal or seizure-like discharges in human subicular slices, underscoring that these rhythms often emerge from a pathologically hyperexcitable network state rather than from intrinsic physiological dynamics (Alvarado-Rojas et al., 2015). Although such ionic manipulations are valuable for probing mechanisms of synchronization and epileptiform activity in human tissue, direct comparisons with in vivo cortical rhythms recorded under intact homeostatic conditions are only limited.

Importantly, previous studies have characterized epileptiform dynamics and ionic mechanisms in human hippocampal and neocortical slice preparations. Changes in the extracellular ionic environment strongly influence network excitability and pathological bursting. An increase in extracellular K^+^ alone, or together with reduced Ca^2+^, reliably induces seizure-like events and synchronous discharges in dentate gyrus, subicular, and neocortical tissue from patients with temporal lobe epilepsy (Antonio et al., 2016; Gabriel et al., 2004). These findings show that ionic manipulations used *in vitro* do not simply create artificial hyperexcitability, but reflect mechanisms that also contribute to seizure generation in the human cortex.

### Consequences of methodological diversity

The heterogeneity in the methodological approaches leads to pronounced variability in oscillation frequency, amplitude, and temporal structure. Table 1 indicates similar nominal categories of oscillations such as “slow oscillations” or “interictal-like activity,” span nearly an order of magnitude in frequency across studies. This variability challenges attempts to classify oscillations into discrete functional categories and raises questions about their physiological equivalence. Differences in slice thickness, chamber type, temperature, ionic composition, and induction protocols often overshadow physiological factors such as brain region or disease state. This limits reproducibility and makes it difficult to meaningfully compare and integrate findings across laboratories (Figure 1D). Without standardized methodological procedures, it remains difficult to determine whether discrepancies across studies reflect genuine biological differences or methodological artifacts.

In addition, variability in methodology complicates the comparison between *ex vivo* and *in vivo* rhythms. While some features such as frequency ranges or pharmacological sensitivity may resemble electroencephalography (EEG) or magnetoencephalography (MEG) oscillations, the strong dependence of slice rhythms on artificial conditions necessitates cautious interpretation. Establishing standardized experimental approaches will be essential for identifying which *ex vivo* oscillations meaningfully reflect intact human network dynamics and for translating slice-level findings to clinically relevant brain states.

## Mechanisms underlying rhythmic network activity

### Role of interneuron subtypes

Inhibitory interneurons play a central role in the generation, pacing, and stabilization of network oscillations. In human cortical and hippocampal slices, multiple interneuron subtypes have been implicated in rhythmic activity, with parvalbumin (PV), somatostatin (SST), and vasoactive intestinal peptide (VIP) interneurons exerting distinct but complementary influences on network dynamics.

PV-expressing interneurons are characterized by fast-spiking action potential (AP) firing patterns and a perisomatic innervation of pyramidal neurons; they are widely regarded as key contributors to fast oscillations, particularly in the γ-frequency range. In hippocampus, histologically verified PV-immunoreactive basket cells often show a broad peak in their auto-correlograms and spectrograms at γ-frequency (Bragin et al., 1995; Buzsaki et al., 2003; Hajos et al., 2004; Mann et al., 2005). Evidence from human neocortical slices indicates that γ-like oscillations are highly sensitive to GABAergic transmission and to manipulations that affect PV interneuron excitability (Roopun et al., 2010). The tight temporal coupling between PV interneuron firing and pyramidal cell spiking supports rapid synchronization across local networks, enabling precise timing relationships that are essential for high-frequency oscillatory activity. Apart from this, inhibition of PV interneurons have been proposed to disrupt a learning-induced increase of δ (0.5–4 Hz) and θ-oscillations (4–10 Hz) in mouse hippocampus (Ognjanovski et al., 2017). During slow oscillations (<1 Hz), PV interneurons were the most active interneuron subtype during cortical up states in rodent neocortex (Neske et al., 2015). Consistent with this, recent evidence from human neocortex further suggests that putative PV-positive large basket cells are preferentially recruited during slow rhythmic network activity and are distinguished by particularly broad and dense dendritic and axonal arborizations, positioning them to exert powerful and widespread inhibitory control over local circuits (Yang et al., 2024).

SST-expressing interneurons constitute a major class of GABAergic neurons that play a distinct and complementary role in the generation and modulation of network oscillations. Unlike PV-expressing interneurons, which are tightly associated with fast rhythmic activity, SST interneurons primarily regulate slow and state-dependent network dynamics through their dense and spatially specific dendritic inhibition of pyramidal neurons. SST interneurons show high levels of spontaneous, tonic firing that persist even in the absence of synaptic input, providing a sustained inhibitory tone that can shape network excitability across extended timescales (Fanselow et al., 2008; Urban-Ciecko et al., 2015). Through the release of GABA acting on both fast GABA_A_ receptors and slow, metabotropic GABA_B_ receptors, SST interneurons are well positioned to influence slow oscillations, up–down state transitions, and the termination of persistent network activity (Neske et al., 2015; Palmer et al., 2012; Urban-Ciecko and Barth, 2016; Urban-Ciecko et al., 2015). SST interneurons of the Martinotti and O-LM cell types, have translaminar axonal projections that preferentially innervate distal dendritic compartments, such as the apical tuft dendrites of pyramidal neurons in cortical layer 1 and the hippocampal stratum lacunosum moleculare (Katona et al., 2014; Packer et al., 2013; Silberberg and Markram, 2007; Wang et al., 2004). These properties allow them to selectively modulate long-range and feedback excitatory inputs. SST INs are dynamically regulated by behavioral state and neuromodulatory systems, including acetylcholine and noradrenaline, which can enhance their activity during specific network states such as θ-oscillations (Chen et al., 2015; Xiang et al., 1998). Electrical coupling via gap junctions further allows specific subsets of SST interneurons to synchronize AP firing, potentially coordinating widespread dendritic inhibition (Gibson et al., 2005; Hu and Agmon, 2015). Together, these properties suggest that SST interneurons do not directly trigger fast oscillations but instead modulate the temporal structure of network activity by gating dendritic integration, regulating slow rhythms, and shaping cross-frequency interactions that link slow oscillatory states to higher-frequency rhythms.

VIP cells, although less directly involved in rhythm generation, exert a powerful modulatory influence through disinhibitory circuits. By selectively inhibiting SST and, to a lesser extent, PV interneurons, VIP cells gate the recruitment of inhibitory networks, thereby dynamically regulating oscillatory gain. In rodent neocortical preparations, activation of VIP cells has been shown to disinhibit local pyramidal neurons and modulate excitatory network activity, particularly in response to cholinergic inputs, suggesting a potential role of neuromodulatory drive in gating synchronized cortical states (Apicella and Marchionni, 2022; Koukouli et al., 2017).

Beyond their individual contributions, interactions among interneuron subtypes are critical for coordinating oscillations across different frequency bands. Cross-frequency coupling, such as θ-γ interactions, is widely observed in hippocampal and cortical circuits and is believed to arise from coordinated interactions between multiple inhibitory cell types. In human neocortex, θ-γ coupling has been reported to persist even under partial synaptic blockade, suggesting that interneuron synchronization may involve both synaptic and non-synaptic mechanisms, including electrical coupling via gap junctions (Roopun et al., 2010). Such findings highlight that interneuron networks in human tissue can sustain coherent oscillatory dynamics through multiple, partially redundant pathways.

### Synaptic and circuit determinants of network oscillations

Oscillatory activity critically depends on the balance between excitatory and inhibitory synaptic transmission, and this principle is strongly supported by observations in human brain slices. Rhythmic network activity usually breaks down when either glutamatergic or GABAergic signaling is perturbed, highlighting that oscillation dependent on balanced and tightly regulated excitation–inhibition (E–I) interactions (Table 1). Hyperexcitable states induced by reduced inhibition or enhanced excitation often give rise to interictal-like discharges and pathological synchronization, whereas excessive inhibition can suppress physiological rhythms altogether. Different frequency bands place distinct demands on synaptic kinetics and receptor composition. Fast oscillations are particularly sensitive to α-amino-3-hydroxy-5-methyl-4-isoxazolepropionic acid (AMPA) receptor–mediated excitation and rapid GABA_A_ receptor–mediated inhibition. AMPA receptors provide the fast excitatory drive required to recruit interneuron networks, whereas GABA_A_ receptor–mediated synaptic currents impose temporally precise inhibitory windows that synchronize pyramidal cell firing (Fuchs et al., 2001; Kujala et al., 2015). In contrast, NMDA receptors play a more prominent role in slower rhythms and sustained network states. In human cortical tissue, NMDA receptor–dependent dendritic integration is enhanced by a low dendritic membrane capacitance which increases synaptic charge transfer and neuronal excitability, reflecting distinctive properties of human pyramidal neurons (Eyal et al., 2016). At the network level, the voltage-dependent activation and slow kinetics of NMDA receptors support prolonged depolarizations, facilitate recurrent excitation, and promote the emergence of synchronous population and interictal-like activity (Cohen et al., 2002; Toth et al., 2018).

Short-term synaptic plasticity further shapes oscillatory activity by dynamically adjusting synaptic efficacy during repetitive activity. Activity-dependent depression at excitatory synapses can constrain runaway excitation and contribute to the termination of oscillations, whereas facilitation at inhibitory synapses may enhance interneuron recruitment during sustained network activity. In human cortical slices, the dynamics of short-term synaptic plasticity differ substantially across neuronal cell types and cortical layers, introducing additional heterogeneity into network behavior. Experimental and theoretical work has shown that short-term synaptic dynamics can regulate the timing, phase relationships, and stability of oscillatory networks by coupling synaptic strength and temporal profile to network frequency (Martinez et al., 2019). These synaptic dynamics influence oscillation frequency, stability, and susceptibility to phase resetting, particularly during transitions between network states (Anwar et al., 2017).

Beyond synaptic mechanisms, cellular and circuit-level organization imposes strong constraints on rhythmic network activity. Previous studies show that a subset of neurons may act as pacemakers or hubs that disproportionately influence network timing. Such neurons often express intrinsic conductances, including persistent Na^+^ and low-threshold Ca^2+^ currents, that generate a slow, regenerative depolarization during the interspike interval and support autonomous rhythmic firing (Bean, 2024; Ramirez et al., 2004). In human slices, layer 5 intrinsically bursting pyramidal neurons have been identified that exhibit rhythmic burst firing at slow and δ frequencies, which persists following blockade of fast glutamatergic transmission and is critically dependent on persistent sodium current, consistent with an intrinsic pacemaker-like mechanism (Tryba et al., 2011). This suggests that a relatively small population of highly connected neurons can play an outsized role in coordinating network rhythms. Oscillatory activity in human slices thus reflects a dynamic equilibrium shaped by synaptic kinetics, receptor composition, and plasticity mechanisms operating across multiple timescales.

### Neuromodulatory modulation of oscillations

Neuromodulatory systems influence rhythmic activity in human brain slices primarily by reshaping synaptic gain and the temporal coordination of inhibition, thereby modulating the expression and stability of network oscillations. Acetylcholine has been shown to broadly regulate network activity across multiple frequency bands, suppressing slow oscillations and cortical up states while promoting θ-rhythms and γ-oscillations through muscarinic- and nicotinic-receptor–dependent mechanisms (Betterton et al., 2017; Howe et al., 2017; Steriade, 1993, 2004; Tukker et al., 2020). Consistent with this, studies in human cortical slices indicate that cholinergic modulation is also highly state dependent: exogenous acetylcholine desynchronizes spontaneous slow network oscillations, supporting a role in shifting cortical activity away from synchronized low-frequency states (Yang et al., 2024). In contrast, activation of muscarinic receptors with carbachol can induce very fast oscillatory activity (>80 Hz) in human neocortex, indicating that cholinergic signaling can also facilitate high-frequency network rhythms under conditions of elevated excitability (Florez et al., 2015). Alongside cholinergic modulation, the noradrenergic system provides an additional layer of state-dependent control over large-scale network dynamics. Via tonic and phasic activity of the locus coeruleus, norepinephrine (NE) has been reported to modulate brain oscillations by shifting cortical and thalamic networks from synchronized slow-wave activity toward desynchronized, high-frequency patterns characteristic of wakefulness, thereby setting arousal state and facilitating sensory and cognitive processing (Berridge et al., 1993; Berridge and Waterhouse, 2003). In anesthetized rats, activation of the locus coeruleus induces hippocampal θ-rhythm (3–6 Hz), whereas reversible inactivation of the locus coeruleus abolishes this rhythm, indicating that in the hippocampus, noradrenergic input is a critical modulator of this form of oscillatory activity (Broncel et al., 2020). *Ex vivo* studies in human cortical slices indicate that norepinephrine can enhance synaptically driven LRDs via activation of *β*₂-adrenergic receptors (Yang et al., 2024). Moreover, although serotonergic and dopaminergic modulation of oscillatory activity has been less extensively characterized in human cortical slices, available evidence from patients and animal models suggests that these systems also act in a cell-type- and receptor-specific manner. Serotonergic signaling modulates oscillatory dynamics, with 5-HT release altering slow and γ oscillations in prefrontal cortex via 5-HT₁A and 5-HT₂A receptors, and influencing γ power in cortical networks linked to cognitive function (Athilingam et al., 2017; Celada et al., 2013; Puig et al., 2010; Sorman et al., 2011). Similarly, dopaminergic modulation influences network rhythms: dopaminergic drugs and basal ganglia circuits are associated with changes in β-band oscillations and functional connectivity across cortico-striatal-thalamo-cortical loops, implicating dopamine in both normal and pathological rhythmic activity (Zhang et al., 2022). Together, these findings highlight that neuromodulatory systems exert convergent yet distinct control over network oscillations, with effects that depend on brain state, receptor engagement, and cellular context.

## Translational and functional implications

One of the most extensively studied applications of human brain slices concerns mechanisms involved in epilepsy. Tissue resected from epileptic patients often exhibits spontaneous or easily inducible pathological rhythmic activity such as interictal-like discharges and hypersynchronous population events. These rhythms reflect an altered excitation–inhibition balance, frequently associated with reduced inhibitory control, enhanced recurrent excitation, or changes in intrinsic membrane properties. Importantly, abnormal oscillatory activity is not confined to the seizure onset zone. Tissue distant from the epileptogenic focus can also display altered rhythmic dynamics, suggesting widespread network reorganization rather than strictly focal pathology. Comparisons between epileptic and non-epileptic cortex in slice preparations reveal both quantitative and qualitative differences in rhythmic activity (Toth et al., 2018). Epileptic tissue tends to show increased network excitability and hypersynchronous activity, manifested as a higher probability of spontaneous population events, larger local field potential and current source density amplitudes, and the exclusive emergence of interictal-like discharges that are absent in non-epileptic cortex. These distinctions emphasize the value of determining readouts of oscillatory activity as functional markers of circuit integrity and excitability.

Human tissue resected from brain tumor patients, including glioblastoma, offers another translationally relevant experimental model system for studying human cortical physiology and pathology. Glioma-adjacent cortex frequently exhibits altered neuronal excitability and abnormal rhythmic activity, even when gross histopathology appears relatively preserved (McAlpine et al., 2025; Montgomery et al., 2020). Tumor-associated changes in extracellular ion concentrations and elevated glutamate levels, impaired neurotransmitter uptake, and dysregulated inhibitory signaling contribute to local hyperexcitability and seizures (Buckingham et al., 2011; Dive et al., 2025; Robert et al., 2015; Tewari et al., 2018). Studying these aberrant rhythmic dynamics will help with explaining the persistence of epilepsy following tumor resection, by revealing durable network-level alterations that outlast removal of the primary lesion.

Together, findings from epileptic and tumor-adjacent tissue highlight a central methodological challenge in human slice research: human “control” tissue is rarely entirely free of underlying pathological influences and subtle disease-related alterations may already bias network rhythms and other mechanisms. In addition to improving pathological and immunohistochemical screening to constrain the degree of covert pathology in human tissue, the field has largely moved away from tryingto obtain truly “healthy” human control tissue. Instead, researchers use relative, disease-matched controls combined with within-patient comparisons, multimodal readouts, and continuum-based models of network dysfunction. The aim is no longer to eliminate bias completely, but to characterize it, constrain it where possible, and incorporate it explicitly into the analysis.

Although epilepsy and brain tumors constitute the main sources of resected human brain tissue, smaller amounts of cortical tissue may also be obtained from surgeries performed for vascular malformations, functional neurosurgical interventions for psychiatric or movement disorders, and, rarely, hydrocephalus or traumatic injury. However, these sources are far less common and often associated with substantial pathological or methodological limitations.

Even with these limitations, human brain slices offer a valuable way to test how pharmacological agents may modulate rhythmic activity in human-specific circuits. Antiepileptic drugs, for example, often dampen pathological oscillations and interictal-like discharges by enhancing inhibition or reducing excitatory drive. Measuring directly in human tissue makes it possible to assess whether candidate compounds have the capacity to normalize network dynamics, without depending entirely on animal models. Importantly, drug effects on physiological rhythms can differ markedly from those observed in animal models, in particular rodents, reflecting species-specific receptor expression and synaptic properties. More generally, measures of oscillatory activity offer a sensitive functional endpoint for pharmacological testing. Changes in rhythm frequency, coherence, or stability can reveal subtle circuit effects that are not apparent at the level of single-cell excitability. In that sense, rhythmic activity can serve as a translational biomarker for evaluating therapeutic efficacy and potential side-effect profiles in a human-relevant context.

Another important reason to study rhythmic activity in human slices is to bridge neuronal microcircuit mechanisms and macroscopic brain signals. Many oscillations observed *ex vivo* fall into the same frequency bands as those measured with EEG or MEG, making it possible to form testable hypotheses about cellular origins of brain rhythms that are observed clinically.

In this manner, slice experiments can provide the basis for an interpretation of non-invasive recordings, for example by linking changes γ-power to specific interneuron dysfunction or synaptic deficits. However, not all rhythmic neuronal activity observed in slices is well suited for translational interpretation. Oscillations that require strongly non-physiological extracellular ionic conditions primarily reflect experimentally induced hyperexcitability rather than normal brain dynamics. Similarly, oscillations that appear only during global GABA_A_ receptor blockade or extreme pharmacological manipulation are best viewed as models of pathological synchronization rather than proxies of physiological rhythms.

In the longer term, combining human slice data with patient-specific clinical information could support for precision medicine. Network oscillations could help stratify patients based on circuit-level phenotypes rather than purely anatomical or genetic criteria. In this approach, human slices may serve as personalized platforms for pharmacological interventions, potentially allowing to measure how an individual’s neuronal circuits respond to specific drugs. Although major substantial technical and ethical challenges remain, this illustrates the unique translational potential of rhythmic network studies in human brain slices.

In summary, rhythmic activity in acute slices from human brain tissue provide a direct view into disease mechanisms, drug effects, and the cellular basis of human brain oscillation. When interpreted with careful attention to methodology and pathophysiology, these oscillations can provide valuable translational insights that complement *in vivo* and clinical studies.
